# Improved 1-year outcomes after active cooling during left atrial radiofrequency ablation

**DOI:** 10.1007/s10840-023-01474-3

**Published:** 2023-01-21

**Authors:** Christopher Joseph, Jose Nazari, Jason Zagrodzky, Babette Brumback, Jacob Sherman, William Zagrodzky, Shane Bailey, Erik Kulstad, Mark Metzl

**Affiliations:** 1 University of Texas Southwestern Medical Center, Dallas, TX 75390 USA; 2 NorthShore University Health System, Evanston, IL USA; 3 Texas Cardiac Arrhythmia Institute, St. David’s South Austin Medical Center, 901 W Ben White Blvd, Austin, TX 78704 USA; 4 Department of Biostatistics, College of Public Health & Health Professions, College of Medicine, University of Florida, Gainesville, USA; 5 Washington University in Saint Louis, 1 Brookings Dr, MO 63130 St. Louis, USA; 6 Colorado College, 14 E Cache La Poudre St, Colorado Springs, CO 80903 USA

**Keywords:** Radiofrequency ablation, Atrial fibrillation, Esophageal cooling, Esophageal protection, Pulmonary vein isolation, Procedural efficacy

## Abstract

**Background:**

Active esophageal cooling during pulmonary vein isolation (PVI) with radiofrequency (RF) ablation for the treatment of atrial fibrillation (AF) is increasingly being utilized to reduce esophageal injury and atrioesophageal fistula formation. Randomized controlled data also show trends towards increased freedom from AF when using active cooling. This study aimed to compare 1-year arrhythmia recurrence rates between patients treated with luminal esophageal temperature (LET) monitoring versus active esophageal cooling during left atrial ablation.

**Method:**

Data from two healthcare systems (including 3 hospitals and 4 electrophysiologists) were reviewed for patient rhythm status at 1-year follow-up after receiving PVI for the treatment of AF. Results were compared between patients receiving active esophageal cooling (ensoETM, Attune Medical, Chicago, IL) and those treated with traditional LET monitoring using Kaplan–Meier estimates.

**Results:**

A total of 513 patients were reviewed; 253 received LET monitoring using either single or multi-sensor temperature probes; and 260 received active cooling. The mean age was 66.8 (SD ± 10) years, and 36.8% were female. Arrhythmias were 60.1% paroxysmal AF, 34.3% persistent AF, and 5.6% long-standing persistent AF, with no significant difference between groups. At 1-year follow-up, KM estimates for freedom from AF were 58.2% for LET-monitored patients and 72.2% for actively cooled patients, for an absolute increase in freedom from AF of 14% with active esophageal cooling (*p* = .03). Adjustment for the confounders of patient age, gender, type of AF, and operator with an inverse probability of treatment weighted Cox proportional hazards model yielded a hazard ratio of 0.6 for the effect of cooling on AF recurrence (*p* = 0.045).

**Conclusions:**

In this first study to date of the association between esophageal protection strategy and long-term efficacy of left atrial RF ablation, a clinically and statistically significant improvement in freedom from atrial arrhythmia at 1 year was found in patients treated with active esophageal cooling when compared to patients who received LET monitoring. More rigorous prospective studies or randomized studies are required to validate the findings of the current study.

## Introduction

Esophageal injury culminating in atrioesophageal fistula (AEF) that occurs weeks after radiofrequency (RF) ablation for the treatment of atrial fibrillation (AF) is one of the most severe complications of pulmonary vein isolation (PVI) [1, 2]. Although rates of AEF are assumed to be low at between 0.1 and 0.4%, the mortality rate is as high as 80% even with early diagnosis and treatment [1, 3]. As such, a number of strategies such as luminal esophageal temperature (LET) monitoring or esophageal deviation have been utilized in attempts to reduce the occurrence of AEF, but most have been shown to be of limited benefit, with some recent studies suggesting trends towards harm [3–5].

Active esophageal cooling was first proposed shortly after the first report of an AEF arising from endocardial ablation [6]. Early data suggested the potential for benefits in reducing esophageal injury even with low-capacity heat extraction from direct liquid instillation [7]. A commercially available esophageal cooling device has demonstrated even greater efficacy at reducing severe esophageal lesions during RF ablation [8, 9], resulting in increased adoption of this approach and a growing number of investigations into its effects on procedural factors [10, 11].

One outcome that has not been evaluated is the effect of active esophageal cooling on long-term procedural outcomes. The earliest mathematical model of esophageal cooling raised the question of whether the use of an expandable cooled balloon in the esophagus could reduce the ability to achieve transmural lesions in the atrium [6]. Newer mathematical models have suggested little influence on atrial tissue temperatures when using a non-expandable active esophageal cooling device [12, 13]. Long-term follow-up data from the largest randomized controlled trial of active esophageal cooling have been published, showing an arrhythmia recurrence of 21.1% in actively cooled patients versus 24.1% in patients receiving LET monitoring [14]. Clinical data have shown that sequential lesions placed immediately after one another have a higher likelihood of achieving transmurality-associated unipolar electrograms compared to delayed lesions [15]. Improved continuity of lesion placement without pauses or relocations of the RF catheter during ablation can improve pulmonary vein isolation success and long-term freedom from arrhythmia [16]. Because active esophageal cooling allows uninterrupted lesion placement without the need to pause for temperature alarms, there is a mechanistic rationale for active esophageal cooling to improve long-term procedural efficacy over LET monitoring. Additionally, recent studies using a phantom gel model highlight the potential harm to the esophagus from uninterrupted heating, which may support the hypothesis that esophageal cooling would be particularly helpful when using uninterrupted ablation technique [17]. With this in mind, we sought to quantify the association between 1-year rhythm status and the type of esophageal protection utilized in a large cohort of patients across two healthcare systems.

## Methods

### Patients

This was a retrospective data review performed with Institutional Review Board approval from the two healthcare systems involved, the NorthShore University HealthSystem and the Texas Cardiac Arrhythmia Research Foundation at St. David’s South Austin Medical Center. A consent form was not required because this was a secondary research that used identifiable private information recorded by the investigator in such a manner that the identity of the human subjects cannot readily be ascertained directly or through identifiers linked to the subjects. All patients who underwent PVI performed by any of four electrophysiologists in two healthcare systems encompassing 3 different hospitals over the study time frame from January 2018 to March 2020 were included in this analysis. No patients who underwent PVI were excluded from this review. The Strengthening the Reporting of Observational Studies in Epidemiology (STROBE) guidelines for reporting observational studies were followed. The time period chosen for this analysis was selected to ensure similarity of treatment across the time period analyzed, with the only significant change being the adoption of active esophageal cooling. The ablation techniques used at each site otherwise remained similar (all patients had high-power short duration (HPSD), using 50 W at one site, 40 W at the other site, with contact-force catheters, using the same ablation index targets before and after adoption of esophageal cooling). As such, the only significant variable to change in this analysis was the use of active esophageal cooling.

### Ablation procedure

The general procedures utilized by operators at the study sites have been previously described [10, 11]. All patients were treated under general anesthesia for their ablation procedure. Electrophysiologist physicians at all sites performed primarily wide area circumferential pulmonary vein isolation with additional posterior wall isolation dependent on physician practice. In all cases, anticoagulation was administered prior to ablation with a heparinized target activated clotting time (ACT) of 300 to 350 s. All sites utilized the Carto system (Biosense Webster, Inc., Diamond Bar, CA, USA) to obtain electroanatomical maps and create a three-dimensional geometry using a mapping catheter (PentaRay or Lasso; Biosense Webster, Inc.). An ICE catheter (AccuNav; Siemens, Mountain View, CA, USA or Soundstar, Biosense Webster, Inc., Diamond Bar, CA, USA) was positioned in the right atrium to guide the transseptal puncture, and for ablation, an externally irrigated ablation catheter (ST/SF™; Biosense Webster, Inc.) was used in all cases, and a steerable decapolar catheter (Webster CS Catheter; Biosense Webster, Inc., Diamond Bar, CA, USA) was placed into the coronary sinus for pacing and recording. A very low to no fluoroscopy protocol was followed for all procedures.

In one healthcare system, the pulmonary veins were isolated by delivery of RF applications circumferentially to the antral regions to produce a minimum of entrance and exit block for at least 20 min, confirmed during isoproterenol infusion at 10 μg/min. A Smartablate™ generator (Biosense Webster, Inc.) was used to deliver RF energy, with a setpoint of 40 W on all patients and all areas of the left atrium. The Surepoint measure (Biosense Webster, Inc.) was utilized during ablations, with a target of 350 units on the posterior wall and 450 units on the anterior wall, lateral wall, and septum.

In the other healthcare system, the posterior wall was isolated using a combination of roof and floor linear lesions, along with additional lesions to further segment the posterior wall to achieve entrance and exit block (using output of 20 mA and 5 ms pulses). The pulmonary veins were isolated by delivery of RF applications circumferentially to the antral regions. The entrance and exit block was confirmed for up to 20 min. A Smartablate™ generator (Biosense Webster, Inc., Diamond Bar, CA, USA) was used to deliver RF energy, with a setpoint of 50 W on all patients and all areas of the left atrium. The Visitag Surpoint module (ablation index) was utilized during ablations, with a target of 400 units on the posterior wall, and 550 units on the anterior wall, lateral wall, and septum and an intertag distance of less than 6 mm. Patients with paroxysmal AF received only pulmonary vein isolation without additional posterior wall ablation, whereas patients with persistent AF received additional posterior wall isolation, in which lines were placed with homogenization until exit block was demonstrated throughout at highest output.

### Esophageal protection

Prior to adoption of active esophageal cooling, standard LET monitoring was utilized in all cases. Adoption of active esophageal cooling occurred in March 2019 in one system and in September 2019 in the other system. In one hospital system, the majority of sensors used before adoption of active esophageal cooling were single-sensor probes, with approximately 10% of them receiving a multi-sensor probe (Circa S-Cath; Circa Scientific, Inc., Englewood, CO, USA). Energy delivery was discontinued when the maximum LET on the single-sensor temperature probe or on any sensor of the multi-sensor probe rose by more than 0.2 °C/s or exceeded 39 °C. In patients treated with active cooling, the position of lesions on the posterior wall was adjusted if needed to avoid ablation directly over the esophagus if the tissue thickness (atrium and esophageal wall) was less than approximately 2 mm as viewed on ICE. Otherwise, the ablation proceeded in a point-to-point fashion uninterrupted by pauses or alarms. In the other healthcare system, LET monitoring was predominantly via a multi-sensor probe (Circa S-Cath, Circa Scientific, Inc., Englewood, CO, USA). RF ablation was stopped and the site of ablation moved to a different distant area of the veins for any alarm over 0.2 °C/s or a temperature exceeding 38.5 °C.

**Fig. 1:**
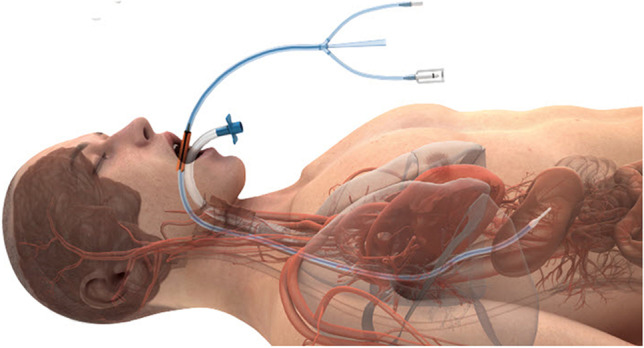
Active esophageal cooling device in place in the esophagus at appropriate depth to ensure cooling behind the left atrium, with endotracheal tube shown in place in the trachea (with permission)

After the adoption of active esophageal cooling in both healthcare systems, no temperature probe was utilized with the esophageal cooling device. Ablation proceeded in a point-to-point fashion uninterrupted by pauses due to temperature elevations or overheating alarms. Active esophageal cooling was performed using the ensoETM device (Attune Medical, Chicago, IL, USA), a closed-loop silicone tube that is inserted into the esophagus in similar fashion to a standard orogastric tube (Fig. 1.). The device is connected to a temperature-controlled heat exchanger which circulates 4 °C water at 2.4 L/min. Placement is confirmed via either fluoroscopy or with ICE. Except for the change to the esophageal cooling protocol, the ablation procedure for patients in each hospital remained the same.

### Definitions

Atrial arrhythmia included AF, atrial flutter, and atrial tachycardia (AT). Paroxysmal AF (PAF) was considered to be episodes of AF that terminate spontaneously. Patients with persistent AF had episodes that continued for > 7 days and were not self-terminating. Patients with long-standing persistent AF had ongoing long-term episodes of AF for over a year.

### Determination of rhythm status

Rhythm status was determined by review of the electronic medical record or existing practice records maintained by the physician practice group. This review focused specifically on status at the 12-month follow-up visit, or the last visit attended by the patient earlier than the 12-month follow-up visit in cases in which a visit was not completed within 2 months of the 12-month anniversary of the index PVI procedure. In the vast majority of cases, visits were in-person, with only a small number of visits occurring via virtual or telehealth visit. Both hospital systems utilize a policy of stopping antiarrhythmics between 0 and 3 months post ablation. Rhythm status was determined by ECG, Holter monitor, wearable patch monitor, pacemaker/defibrillator, or implantable loop recorder. Wearable patch monitoring was typically performed with commercially available devices such as Zio patch (iRhythm Technologies, San Francisco, CA) or CAM patch (Bardy Diagnostics, Bellevue, WA). Patient self-monitoring using personal devices, such as AliveCor Kardia, Fitbit, and Apple Watch, also generally increased during this study period, providing another means by which patients might identify a recurrence of arrhythmia and present to the hospital.

### Statistical analysis

Continuous variables are expressed as mean ± standard deviation, with categorical variables reported in percentile value. Kaplan–Meier curves were created and analyzed using the log-rank test to compare the rate of freedom from arrhythmia between patients receiving LET monitoring and patients receiving active esophageal cooling. To adjust for confounders, we used inverse probability of treatment weighted estimates of a Cox proportional hazards ratio [18]. These weights are constructed as the reciprocal of the probability of received treatment (cooling versus control) modeled as a logistic regression of cooling on the confounders. The confounders are balanced in the weighted sample. We used the Hosmer–Lemeshow test to assess model fit for the logistic regression. There are likely secular trends in AF incidence due to increased use of wearable monitors over time and the greater age and comorbidities of patients undergoing ablations; however, these trends produce bias towards the null. Results were considered significant with a *p* value < 0.05. Statistical analyses were performed using IBM SPSS Statistics software, version 26 (IBM, Armonk, NY).

## Results

A total of 513 patients were reviewed; 253 received LET monitoring using either single or multi-sensor temperature probes; and 260 received active cooling. The mean age was 66.5 ± 9.7 in the LET-monitored group and 66.9 ± 9.8 in the actively cooled group (Table 1). Gender was 63% male in the LET-monitored group and 64.6% male in the actively cooled group. Arrhythmias were 59.7% paroxysmal AF, 35.6% persistent AF, and 4.7% long-standing persistent AF in the LET-monitored group, and 60.4% paroxysmal AF, 33.1% persistent AF, and 6.5% long-standing persistent AF in the actively cooled group.

**Table 1:** Patient demographics and type of atrial fibrillation

	LET monitoring (*n* = 253)	Active esophageal cooling (*n* = 260)	*p* value
Patient age (years)	66.5 ± 9.7	66.9 ± 9.8	0.64
Age range (years)	21.1 to 88.0	32.0 to 91.3	
Gender (% male)	63.0	64.6	0.70
AF type			
Paroxysmal, *n* (%)	151 (59.7)	157 (60.4)	0.87
Persistent, *n* (%)	90 (35.6)	86 (33.1)	0.55
Long-standing, *n* (%)	12 (4.7)	17 (6.5)	0.37

In line with typical practice in the USA currently, complete isolation is attained in essentially all patients before considering the case complete and emerging the patient from anesthesia. No differences in this measure have been seen with the use of active esophageal cooling, which reflects what has been reported in randomized controlled studies [14].

The exact method utilized for arrhythmia detection is available for approximately two-thirds of the patients, with the breakdown of method as shown in Table 2. One trend evident from this table is the increased use of more sensitive detection instruments, such as 7-day monitors, Kardia monitor, and Apple watch, which would be expected to increase detection of arrhythmias in the actively cooled group [19–21].

**Table 2:** Methods used to determine arrhythmia recurrence

Method	LET-monitored group (*n* = 98)	Actively cooled group (*n* = 231)
EKG	79.6%	79.2%
Pacer	7.1%	2.2%
ICD	4.1%	2.2%
Apple watch	2.0%	2.2%
Kardia	2.0%	5.2%
Telemetry monitor	2.0%	0.9%
7-day monitor	1.0%	2.2%
Echocardiogram	1.0%	0.9%
LINQ™	1.0%	0.0%
Blood pressure machine	0.0%	0.4%
Clinical symptoms	0.0%	2.2%
EP study	0.0%	2.2%
During surgery	0.0%	0.4%

Abbreviation: LINQ, Reveal LINQ insertable cardiac monitor (ICM)

Specific antiarrhythmic drugs used in this subset are as shown in Table 3.

**Table 3:** Antiarrhythmic drug use in patients

Antiarrhythmic drug	LET-monitored group (n = 98)	Actively cooled group (*n* = 231)	p value
Amiodarone	5.1%	10.0%	0.15
Dofetilide	2.0%	0.9%	0.37
Dronedarone	1.0%	1.7%	0.63
Flecainide	3.1%	3.0%	0.99
None	87.8%	82.7%	0.25
PRN flecainide	0.0%	0.4%	0.52
Sotalol	1.0%	1.3%	0.83

Additional patient-level factors, such as left atrial size, CHA2DS2-VASc Scores, re-do ablations, and antiarrhythmic drug use, were available for only a subset of patients, representing approximately one-third of the total (180 patients), in this review. For this subset, the mean left atrial size was 4.29 cm (SD 0.75 cm), mean CHA2DS2-VASc score was 2.46 (SD 1.55), and re-do ablation rate was 22.1%.

**Fig. 2:**
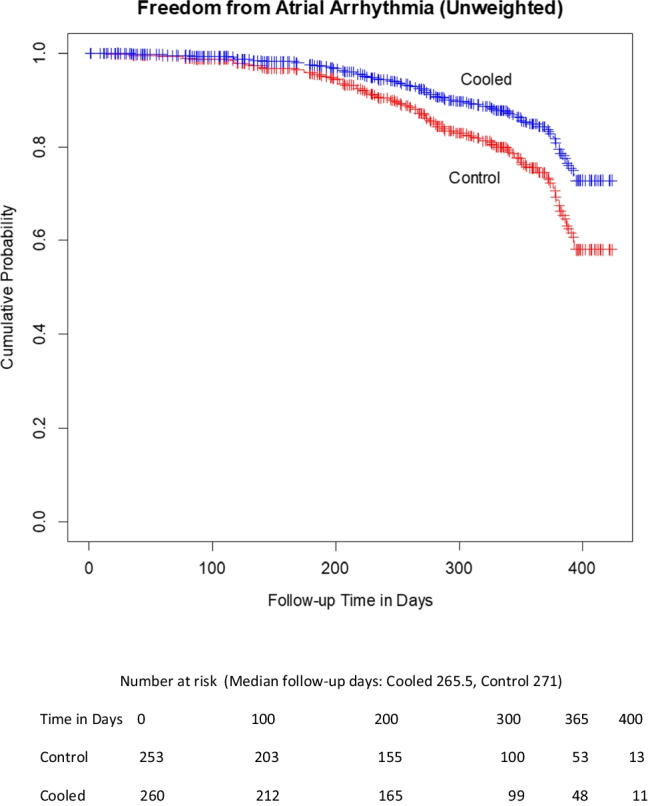
Unadjusted Kaplan–Meier estimates of freedom from arrhythmia. The rate of freedom from arrhythmia was significantly higher for patients treated with active esophageal cooling, with curves compared using the log-rank test

At 1-year follow-up, KM estimates for freedom from AF were 58.2% for LET-monitored patients and 72.2% for actively cooled patients (Fig. 2), for an absolute increase in freedom from AF of 14% with active esophageal cooling (*p* = 0.03, log-rank).

**Fig. 3:**
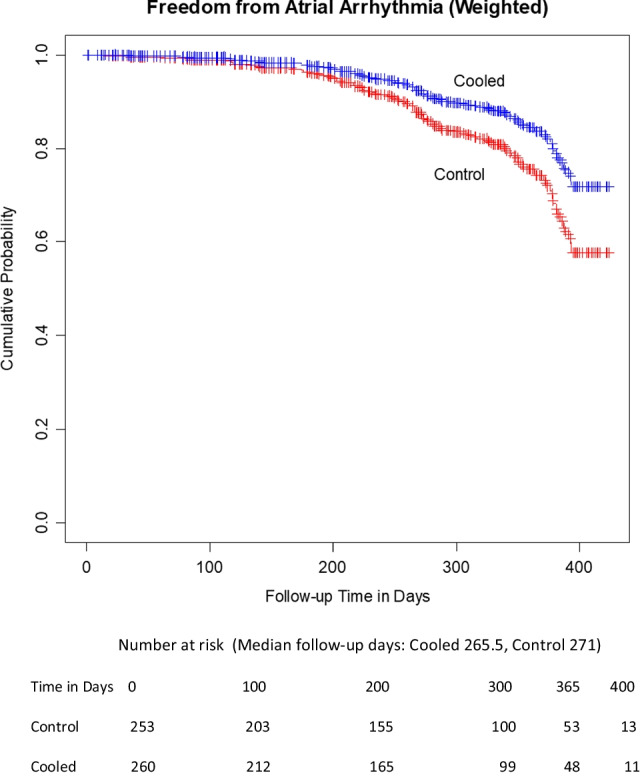
Adjusted estimates of freedom from arrhythmia, using inverse probability of treatment weighting with a weighted Cox proportional hazards model

Multivariate analyses were conducted by computing propensity scores for cooling using a logistic regression of cooling on the specific operator, the type of atrial fibrillation, patient age, and patient gender. Then, inverse probability of treatment weighting was used with a weighted Cox proportional hazards model [18]. The addition of these factors results in an estimate that cooling reduces the hazard of recurrence by a factor of 0.60 (*p* = 0.045), as shown in Fig. 3. Goodness-of-fit for the logistic regression was ascertained using the Hosmer–Lemeshow test (*p* = 0.48). Table 4 shows the logistic regression model parameter estimates for the probability of treatment weights.

**Table 4:** Logistic regression model parameter estimates for the probability of treatment weights

Confounder	Odds ratio	95% CI
Age	1.0039	(0.985, 1.023)
Male	1.0	NA
Female	0.981	(0.671, 1.434)
Paroxysmal AF	1.0	NA
Persistent AF	1.106	(0.763, 1.608)
Operator 1	1.0	NA
Operator 2	0.394	(0.161, 0.950)
Operator 3	1.132	(0.488, 2.608)
Operator 4	1.433	(0.604, 3.384)

## Discussion

In this first formal study to date the association between esophageal protection strategy and long-term efficacy of left atrial RF ablation, we found a clinically and statistically significant improvement in freedom from atrial arrhythmia at 1 year in patients treated with active esophageal cooling when compared to patients who received LET monitoring. Previous randomized controlled trial data have suggested trends towards greater freedom from atrial arrhythmias at long-term follow-up, but the smaller number of patients evaluated did not reach statistical significance [14]. Moreover, electrophysiology fellows in training performed the majority of procedures in randomized controlled trials, introducing additional potential procedural inefficiencies. The inclusion of a large number of patients across multiple hospitals with experienced attending electrophysiologists in this analysis increases the robustness and reliability of the findings while demonstrating an important outcome effect associated with active esophageal cooling. All electrophysiologists included in this analysis have greater than 10-year-experience post fellowship (ranging from as little as 10 years to as great as 30 years of experience after fellowship).

**Fig. 4:**
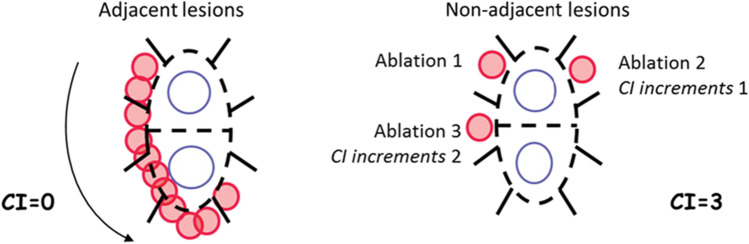
Example of continuity index calculation for two examples of ablation patterns (with permission) [16]

A mechanistic underpinning exists to explain these findings, with previous studies demonstrating the importance of lesion sequence and timing in ensuring complete isolation of the pulmonary veins after a PVI procedure. In the EFFICAS-II study, a formal measurement of lesion placement contiguity was defined as the continuity index (CI) [16]. The CI is quantified by the number of positions in the left atrium that the RF catheter tip is moved over after prematurely ceasing RF power and moving elsewhere in the atrium to perform subsequent ablations in non-adjacent positions (Fig. 4). In general, movement away from a lesion site is compelled by the development of overheating at the site, triggering a temperature alarm or an elevation in temperature that compels the operator to cease delivering RF power. The operator can then either wait for equilibration in temperature or move to a distant site to begin ablations elsewhere in the atrium before returning to the site adjacent to the overheated site to continue the sequence of lesions. In either case, the prematurely aborted lesion is left incompletely transmural, while the adjacent site becomes increasingly edematous from the nearby thermal insult, rendering the new site more difficult to obtain a transmural lesion. The result of this effect is a larger number of partially formed, non-transmural lesions, a reduction in the ability to fully isolate the pulmonary veins, and a decrease in overall efficacy of the procedure. In the EFFICAS-II study, PVI performed with a low CI (CI < 6) had a 98% (56/57) chance of remaining isolated, while PVI performed with a high CI (CI ≥ 6) had only a 62% (21/34) chance of remaining isolated [16].

More recently, clinical data have shown that lesion placement order is an important factor in successful lesion formation [15]. Jankelson et al. reviewed 437,760 position data points during lesion placement, and found that sequential lesions placed immediately after one another have a higher likelihood of achieving transmurality-associated unipolar electrograms compared to delayed lesions [15]. The authors propose explanations for these findings that include the stacking of heat from prior ablation with sequential ablation, and the development of localized edema after lesion placement, resulting in impaired lesion formation with delayed lesions, while noting that edema formation is time dependent and may impede the recording of local electrograms and, thus, may play a greater role with increased temporal delay between lesions [15, 22–27]. As such, these findings provide insight regarding possible mechanisms by which sequential ablation with minimal interruption has been associated with improved ablation outcomes [28].

Active esophageal cooling is performed without the need for temperature sensors, as the esophageal cooling device maintains a coolant temperature of 4 °C, and reduces the potential for esophageal wall temperatures to reach lethal isotherm levels [13, 29]. As a result, premature cessation of RF energy deposition during lesion placement is unnecessary, and uninterrupted lesion placement can be performed without the need to pause for temperature alarms. Thus, a low CI can be maintained throughout the procedure, with most cases resulting in a CI well under 6, and instead commonly equal to zero (most cases in the actively cooled cohort proceeded point-to-point without need for premature cessation of RF energy, yielding a CI of zero, whereas almost all cases with temperature monitoring involved inevitable pauses from temperature alarms). This difference likely correlates with a higher efficacy of isolation of the pulmonary veins, and a resultant improvement in freedom from arrhythmia, when using active esophageal cooling. Randomized controlled data also point to this effect, with the 1-year follow-up of patients in the IMPACT study noting that the presence of the esophageal cooling device permitted the operators to operate more comfortably in constructing lesion sets in the posterior left atrium [14]. The authors found a steady progression from one lesion site to the next, adhering in most cases to the desired 4–6 mm between consecutive lesions (compared to the LET-monitored group in which juddering progress was evident with more instances of movement across a distance of more than 15 mm within the posterior region) [14]. 

## Limitations

This was a retrospective review of data from patients receiving standard of care outside of a formal randomized clinical trial. As such, unmeasured confounders may exist. Other than the change in esophageal protection strategy, no major changes in ablation protocol occurred, and the general characteristics of patients served by the hospitals did not change during the analysis time frame. The study also did not include data on common comorbidities which are known risk factors for AF such as patient BMI, HTN, OSA, CHF, and the presence/severity of AF. Further, LA size, CHA2DS2-VASc scores, and the use of AADs were only available for a subset of patients. As such, potential differences in these known risks factors between groups were not accounted for in the multivariate model, and potential bias in the primary outcome between groups cannot be excluded in the current study. Patients receiving re-do ablations were included in this study. Over the time frame analyzed, re-do ablation rates were similar. Another important limitation of the current study was that implantable loop recorders were not utilized uniformly to assess AF burden on follow-up and the study relied on only conventional methods for follow-up. However, in the latter study period, there was an increased utilization of retail ECG monitoring devices and also increased clinical use of wearable monitors by study patients, which may have actually increased sensitivity for arrhythmia detection in the more contemporary active esophageal cooling group [19–21]. Finally, it should be noted that this analysis did not include a 90-day blanking period, and arrhythmia recurrences occurring in this time were included. Recent studies support that early recurrence of AF is a clinically significant predictor of late recurrence of AF post ablation, and thus, failures within the 90-day blanking period were included to improve the robustness of our data [30–34].

## Conclusions

In this first study to date of the association between esophageal protection strategy and long-term efficacy of left atrial RF ablation, a clinically and statistically significant improvement in freedom from atrial arrhythmia at 1 year was found in patients treated with active esophageal cooling when compared to patients who received LET monitoring. More rigorous prospective studies or randomized studies are required to validate the findings of the current study.

## Data Availability

Data available on request from the authors.
